# Dynorphin/kappa opioid receptor system regulation on amygdaloid circuitry: Implications for neuropsychiatric disorders

**DOI:** 10.3389/fnsys.2022.963691

**Published:** 2022-10-05

**Authors:** Aaron Limoges, Hector E. Yarur, Hugo A. Tejeda

**Affiliations:** ^1^Unit on Neuromodulation and Synaptic Integration, Bethesda, MD, United States; ^2^NIH-Columbia University Individual Graduate Partnership Program, National Institutes of Health, Bethesda, MD, United States; ^3^Department of Biological Sciences, Columbia University, New York, NY, United States

**Keywords:** basolateral amygdala, central nucleus of amygdala, dynorphin, kappa-opioid receptor (KOR), G-protein coupled receptors, stress, anxiety, addiction

## Abstract

Amygdaloid circuits are involved in a variety of emotional and motivation-related behaviors and are impacted by stress. The amygdala expresses several neuromodulatory systems, including opioid peptides and their receptors. The Dynorphin (Dyn)/kappa opioid receptor (KOR) system has been implicated in the processing of emotional and stress-related information and is expressed in brain areas involved in stress and motivation. Dysregulation of the Dyn/KOR system has also been implicated in various neuropsychiatric disorders. However, there is limited information about the role of the Dyn/KOR system in regulating amygdala circuitry. Here, we review the literature on the (1) basic anatomy of the amygdala, (2) functional regulation of synaptic transmission by the Dyn/KOR system, (3) anatomical architecture and function of the Dyn/KOR system in the amygdala, (4) regulation of amygdala-dependent behaviors by the Dyn/KOR system, and (5) future directions for the field. Future work investigating how the Dyn/KOR system shapes a wide range of amygdala-related behaviors will be required to increase our understanding of underlying circuitry modulation by the Dyn/KOR system. We anticipate that continued focus on the amygdala Dyn/KOR system will also elucidate novel ways to target the Dyn/KOR system to treat neuropsychiatric disorders.

## Dynorphin/kappa opioid receptor signaling overview

### Translational/clinical significance of dynorphin/kappa opioid receptor signaling

The neuropeptide dynorphin (Dyn) and its cognate receptor, the kappa opioid receptor (KOR), have been implicated in maladaptive behaviors associated with several psychiatric disorders. The Dyn/KOR system constitutes a class of opioidergic signaling that is in large part distinct from other opioid systems such as the mu-or delta-opioid receptor system. In humans, stimulation of KORs drives anxiety, dysphoria, and psychotomimesis (Pfeiffer et al., 1986). These behavioral effects have been observed with various KOR agonists, including the naturally occurring KOR agonist Salvinorin A (Tejeda and Bonci, 2019). Salvinorin A is the primary psychoactive compound in *Salvia divinorum*, a hallucinogenic plant. KOR antagonists have been investigated in the clinic primarily as potential treatments for mood and substance use disorders (Carlezon and Krystal, 2016; Fava et al., 2020) but see Jacobson et al. (2020). Selective antagonism of KORs in humans alleviates symptoms of anhedonia in transdiagnostic studies (Pizzagalli et al., 2020), with a corresponding rescue of activation of the ventral striatum in anticipation of reward delivery (Krystal et al., 2020). Similarly, in rodents, KOR activation with endogenous Dyn or exogenous agonists promotes anxiety-like behavior, aversion, and anhedonia, impairs social interactions, and drives deficits in active coping in response to stressors [reviewed in Bruchas et al. (2010), Wee and Koob (2010), Tejeda et al. (2012), Crowley and Kash (2015), Karkhanis and Al-Hasani (2020)]. It has been speculated that over short periods, Dyn-mediated agonism of KOR may act as an acute punisher to reduce the seeking for other drugs and reinforcers (Freeman et al., 2014; Butelman and Kreek, 2015; Heinsbroek et al., 2018), but over the long term, it may act as a negative reinforcer of such behaviors (Bruchas et al., 2010; Wee and Koob, 2010; Walker et al., 2012; Chartoff et al., 2016; Escobar et al., 2020). Consistent with effects produced by synthetic KOR agonists, the recreational drug salvinorin A promotes similar behavioral effects and blocks the reinforcing effects of other drugs, most notably psychostimulants (Gonzalez et al., 2006; dos Santos et al., 2014; Brito-da-Costa et al., 2021). Salvinorin A, unlike other hallucinogens, does not bind the 5-HT2A receptor (Sheffler and Roth, 2003). Ketamine, a recreational and rapid-acting antidepressant, has also been used as a means to model certain domains of schizophrenia and other dissociative disorders in animal models (Moghaddam and Jackson, 2003; Frohlich and Van Horn, 2014; Beck et al., 2020; Schmack et al., 2021). Ketamine antagonizes NMDA receptors, as well as KORs (Nemeth et al., 2010; Bonaventura et al., 2021). However, it is currently unclear whether direct actions of ketamine on KOR mediates any of the behavioral effects produced by ketamine. The Dyn/KOR system interacts with other stress-related neuropeptide systems, including corticotropin-releasing factor (CRF), which is enriched in neuronal circuits that control affect and motivation (e.g., the central nucleus of the amygdala). Dyn/KOR interactions with CRF contribute to dysregulation of innate and learned fear responses relevant to anxiety-like behavior and affect.

The effects of Dyn/KOR agonism by exogenous or endogenous agonists in animal models result in affective, motivational, and cognitive phenotypes relevant to psychiatric disorders including PTSD, depression, schizophrenia, and substance use disorder. Indeed, such disorders have been associated with alterations in Dyn/KOR expression or function [see Hang et al. (2015), Bruchas et al. (2010), Jacobson et al. (2020) for reviews on this topic]. In psychotic disorders, such as schizophrenia, altered Dyn/KOR signaling may be one factor that contributes to the dysfunction of dopaminergic transmission in mesolimbic and mesocortical circuitry, which mediate various features of positive and negative symptoms, and cognitive deficits [see Tejeda et al. (2012), Clark and Abi-Dargham (2019)]. The Dyn/KOR system may also be involved in substance use disorder, potentially contributing to the development of pro-addictive behaviors during stressful experiences [see Bruchas et al. (2010)]. In mice, stress is also thought to promote alcohol-and drug-seeking behaviors through Dyn/KOR interactions with the CRF system [see Bruchas et al. (2010), Wee and Koob (2010), Walker et al. (2012), Anderson and Becker (2017), Karkhanis and Al-Hasani (2020)]. Together, this highlights a role for Dyn/KOR activation during aversive and stressful experiences and underscores its potential to contribute to psychiatric dysfunction in humans.

The Dyn/KOR system regulates stress-related and goal-directed behaviors *via* actions in circuits that subserve the aforementioned behaviors. Early studies identified that the Dyn/KOR system is embedded in the substantia nigra and the ventral tegmental area, nucleus accumbens (NAcc), amygdala, hypothalamus, paraventricular thalamus (PVT), hippocampus, and septum, and caudate/putamen (Chavkin et al., 1985; Fallon et al., 1985; Slater and Cross, 1986; Mansour et al., 1987; Rattan et al., 1992; DePaoli et al., 1994; Jamensky and Gianoulakis, 1997; Chou et al., 2001). These regions are part of an interconnected limbic network that control various facets of learning/memory, goal-directed behavior, stress, arousal, attention, and energy homeostasis. The Dyn/KOR system is also found in multiple cortical areas including the auditory cortex (Ramsdell and Meador-Woodruff, 1993), the somatosensory cortex (Loh et al., 2017), periamygdaloid cortex (Anderson et al., 2013), and the parietal cortex (DePaoli et al., 1994). Prodynorphin-expressing neurons have also been identified in mouse and human brainstem (Agostinelli et al., 2021), as well as the lateral parabrachial nucleus (Chiang et al., 2020; Norris et al., 2021).

In the vein of translational considerations, there have also been reports of sex differences in the Dyn/KOR system. A PET imaging study detected increased KOR-selective tracer in healthy human males relative to healthy females (Vijay et al., 2016). Multiple single nucleotide polymorphisms (SNPs) in the prodynorphin gene have also been associated with differential susceptibility to development of opioid dependence, with the overall risk of each SNP often differing between sexes and across ethnic populations (Chartoff and Mavrikaki, 2015). The Dyn/KOR system may also be regulated through hormonal signaling, either through direct or indirect regulation of transcription factor binding or other signaling pathways (Chartoff and Mavrikaki, 2015). For example, in the mouse spinal cord, KORs heterodimerize with MORs in a sex-dependent manner, which is regulated by estrogen signaling (Chakrabarti et al., 2010; Liu et al., 2011). The extent to which KOR/MOR heterodimerization occurs in the human brain is still largely unknown, however, thus highlighting the need for additional studies on sex-dependent regulation of this system.

### Mechanisms of circuit neuromodulation by dynorphin and kappa opioid receptor

Ultrastructural evidence shows KOR immunoreactivity within dendritic spines and axon terminals. These results provide an anatomical substrate by which KOR activation regulates the presynaptic release and postsynaptic neuron activity (Drake et al., 1996; Svingos et al., 1999; Svingos and Colago, 2002), to ultimately impact the activity of limbic circuits. KOR is a G-protein coupled receptor (GPCR), coupled to inhibitory G_*i/o*_ proteins to decrease the membrane excitability *via* activation of G-protein gated inwardly rectifying potassium channels (Kir3 family). The activation of GIRK causes cellular hyperpolarization and inhibits neural activity (Torrecilla et al., 2002; Margolis et al., 2003; Ford et al., 2007; Chen et al., 2015). Besides Kir3 activation, KOR activation inhibits Ca^2+^ currents mediated by P/Q-type, N-type, and L-type channels to reduce calcium conductance and/or interfere with presynaptic release machinery downstream of Ca^2+^ entry (Grudt and Williams, 1993; Castillo et al., 1996; Simmons and Chavkin, 1996; Rusin et al., 1997; Hjelmstad and Fields, 2003; Iremonger and Bains, 2009; Tejeda et al., 2017). The impact of Dyn/KOR signaling may be complex depending on how this system is integrated into circuits. For example, Dyn/KOR signaling inhibits glutamate release in the NAcc from specific excitatory inputs, in addition to acting on local inhibitory connections from KOR-expressing accumbal medium-sized spiny neurons. The direct inhibitory effects of KOR signaling on presynaptic inputs filters glutamate release from incoming KOR-sensitive inputs and any influence those inputs may have on post-synaptic activity. Conversely, KOR acting on local circuit collaterals disinhibits other MSNs and facilitates the integration of incoming excitatory input from KOR-lacking afferent inputs (Tejeda et al., 2017). In summary, the available evidence indicates that the KORs are localized on axon terminals as well as on neuronal cell bodies to modulate the activity of the presynaptic compartment or the neuronal activity acting on the postsynaptic cell. A deep understanding of how KORs are embedded within circuits (e.g., on presynaptic vs. post-synaptic compartments, excitatory vs. inhibitory cells, etc.) is essential to deconstruct how the Dyn/KOR system regulates affect and motivation *via* its actions in limbic structures, including the amygdala.

## Amygdala overview

Functional KORs and Dyn peptides have been described in the amygdala, an area which, in humans and other mammals, is critical for cognitive and emotional processing, learning and memory (LeDoux, 2000; Phelps, 2006). Amygdala dysfunction has been implicated in mediating symptomology in a host of psychiatric conditions. Often described as a hub for learning and memory and regulating affective states, the amygdala, like the Dyn/KOR system, is recruited during motivationally-charged experiences, including those associated with physiological or psychological stress (Roozendaal et al., 2009; Janak and Tye, 2015). It is also a region associated with pronounced changes following exposure to stress or stress-related hormones (McEwen, 2007; Roozendaal et al., 2009) and Dyn/KOR signaling within amygdala circuitry may be a key player in this process. Humans bearing the gene polymorphism (T) allele of prodynorphin at rs1997794 show impaired fear extinction and significant decreases in functional connectivity between the amygdala and PFC (Bilkei-Gorzo et al., 2012). As such, the Dyn/KOR system within the amygdala may serve as an interface through which stressors and noxious signals modulate key behavioral and affective states.

Importantly, key populations of excitatory and inhibitory neurons alike have been reported to be largely similar across humans and mice. One study performed a single-nucleus expression profiling of human amygdala and compared these results with a previous profiling study on mouse amygdala, finding that just 10.4% of detected genes were human-specific (Tran et al., 2021). A separate study found that sexual dimorphism in gene expression is largely conserved across human and mice (Lin et al., 2011). Additional controlled studies are needed to further characterize the peptidergic cell types of the human amygdala and contrast them with other species, especially given that the conventional notion of “cell type” has become harder to define in the era of transcriptomics (Yuste et al., 2020). Similarly, investigations on how Dyn/KOR expression, signaling, and/or modulation of amygdala circuit function changes across development are warranted. Research in this area may elucidate how neurodevelopmental challenges, such as early life stress, may contribute to dysfunction of limbic circuits and/or the function of the Dyn/KOR system in promoting maladaptive behaviors in adulthood in patients with psychiatric disorders.

### Functions of the amygdala in behavior

Amygdala circuits control multiple domains of behavior and contribute to cognitive, affective, and social processing. Situated between the cortex and deeper brain regions, connectivity of the amygdala suggests it may serve to integrate incoming sensory streams with state-and experience-dependent information to guide behavior (Sah et al., 2003; Pape and Pare, 2010; Janak and Tye, 2015). The amygdala has been studied extensively as a brain region critical for associative learning for stimuli of both positive and negative valence [see LeDoux (2007), Calu et al. (2010), Pape and Pare (2010), Janak and Tye (2015), Namburi et al. (2015), Kim et al. (2016), O’Neill et al. (2018), Kong and Zweifel (2021)]. Furthermore, the role of the amygdala extends beyond acquisition, as it is also critical for the extinction of associative memories, similarly processing conditioned stimuli of both positive and negative valence (Maren and Quirk, 2004; Tye et al., 2010; Zhang et al., 2020; Whittle et al., 2021). Cognitive flexibility and decision-making are also impacted by amygdala function (Keefer et al., 2021). A recent study also showed the amygdala processes cue contingencies and motivational states to help select behavioral responses under a range of environmental and internal state demands (Courtin et al., 2022). In addition to learning and memory, the amygdala regulates facets of affective behavior (Gallagher and Chiba, 1996). Amygdala dysfunction has been linked to major depressive disorder (MDD) in humans (Nestler et al., 2002), and in mice may also play a role in governing anhedonia-like phenotypes (Ramirez S. et al., 2015). Similarly, because anxiety and depression often co-occur (Britton et al., 2011; Tiller, 2013), it is possible that dysregulated amygdala activity can contribute to both of these conditions (He et al., 2019; Espinoza Oyarce et al., 2020).

Alterations in amygdala activity have been linked to post-traumatic stress disorder (PTSD) as well as substance use disorders in humans (Grillon et al., 1996; Sharp, 2017; Morey et al., 2020; Zhang W.H. et al., 2021; Alexandra Kredlow et al., 2022). Following its role in learning, memory, and affect, especially in the context of psychiatric conditions such as PTSD, the amygdala is also susceptible to stress and itself regulates components of stress processing (Zhang W.H. et al., 2021). As previously mentioned, stress produces lasting changes in amygdala circuitry and connectivity (McEwen, 2007; Roozendaal et al., 2009; Zhang et al., 2019), and the amygdala contains neural populations that express and release CRF (Gray and Bingaman, 1996; George and Koob, 2010; Koob et al., 2014; Zorrilla et al., 2014; Marcinkiewcz et al., 2016). CRF in turn activates other stress-associated neuromodulatory systems in the amygdala, namely cell populations expressing norepinephrine (NE) and dynorphin (George and Koob, 2010; Knoll and Carlezon, 2010; Koob et al., 2014). Together, these studies suggest that changes in stress-related signaling perturb amygdala function and may promote behavioral reactions in response to stressors or gate active behaviors that promote avoidance or escape from stressors and threats.

Overall, the precise mechanisms through which the amygdala governs diverse behaviors are complex. Recently there have been significant advances in understanding the cell types and amygdala microcircuits and long-range interactions that permit amygdala circuitry to control cognitive, emotional, and social behaviors. Studies such as these are necessary to resolve questions on how or why the amygdala specifies affective states such as anxiety and depression as well as cognitive tasks such as learning and extinction.

### Amygdala subregions and circuitry

The amygdala comprises several subregions which vary in their connectivity, cellular subtypes, and function. This includes the central nucleus of the amygdala, basal, and lateral nuclei of the amygdala, and the intercalated cell masses (ITCs). The bulk of this review will focus primarily on the basolateral amygdala (BLA) and central amygdala (CeA), which are two of the most characterized regions of the amygdala.

**FIGURE 1:**
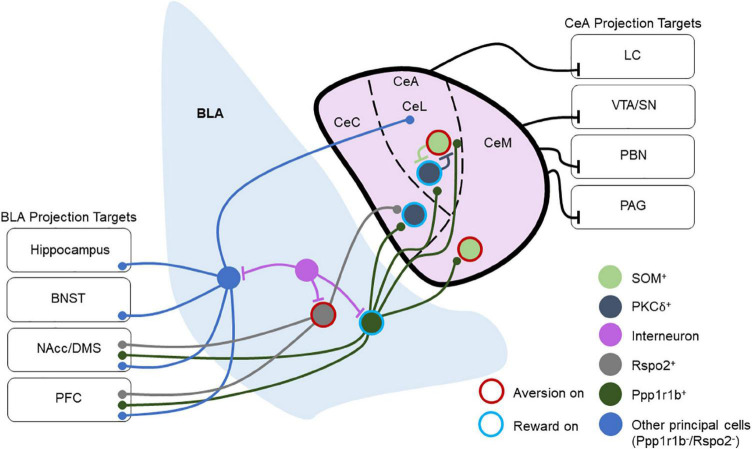
Overview of basolateral amygdala (BLA) and central amygdala (CeA) connectivity and projection targets. This depicts a layout of multiple BLA and CeA cell types and their connectivity schemes in local and long-range circuitry. Cells are labeled as “Aversion on” or “Reward on” that has been tested experimentally. Ppp1r1b^+^ BLA neurons target PKCδ^+^ and SOM^+^ neurons throughout the CeA, and they also project to the NAcc and PFC. Rspo2^+^ BLA neurons target PKCδ^+^ neurons in the CeC and send projections to the NAcc and PFC. Other BLA principal neurons project to the CeA as well as to several other brain regions including the hippocampus, BNST, NAcc, DMS, and PFC. Within the CeL, SOM^+^, and PKCδ^+^ populations reciprocally inhibit one another. CeM neurons also send long-range inhibitory projections to the VTA, SN, PBN, and PAG, while the CeL sends long-range inhibitory projections to the LC. Through these projections, the CeA regulates arousal, attention, movement, and defensive behaviors.

Most hypotheses of amygdala function contend that the BLA integrates the temporal structure of sensory and state information before passing it to other regions, such as the downstream CeA, through glutamatergic projection neurons. The excitatory inputs from BLA target both the lateral and medial compartments of the CeA, with innervation of the lateral compartment coming primarily from the LA and innervation of the medial compartment from the BA. The lateral nucleus of the CeA (CeL) also targets the medial nucleus of the CeA (CeM), but reciprocal projections from CeM to CeL have not been observed. Importantly, the CeL is involved in fear acquisition, while the CeM is critical for fear expression and extinction (Ciocchi et al., 2010; Haubensak et al., 2010). A third subregion of the CeA is the capsular region, although this area is less well-studied (Figure 1).

Recent studies have refined the model of BLA-CeA processing by investigating the effects of this pathway on appetitive behaviors. Kim et al. found that genetically distinct populations of BLA neurons target specific populations of CeA neurons that are responsible for appetitive or aversive behaviors (Kim et al., 2017).

### Basolateral amygdala inputs and outputs

The regions supplying the densest afferents to the BLA include the thalamus, ventral hippocampus, dorsal raphe nucleus, posterior intralaminar nucleus, medial geniculate nucleus, ventral tegmental area, nucleus accumbens, and cortical areas including the prefrontal cortical areas and other sensory cortical regions (Steinbusch, 1981; Bocchio et al., 2016; Fu et al., 2020; Hintiryan et al., 2021; Morikawa et al., 2021). Each of these input pathways relays different forms of state, motivational, or sensory information to the BLA. Synaptic integration of these converging pathways into the BLA is regulated by neuromodulators, including monoamines and neuropeptides, *via* target receptors in the BLA and presynaptic terminals from afferent inputs.

Outputs of the BLA include the CeA, BNST, lateral hypothalamus (LH), nucleus accumbens, ventral tegmental area, and the BLA’s reciprocal connections with the mPFC and ventral hippocampus (Pape and Pare, 2010; Janak and Tye, 2015; Hintiryan et al., 2021; Murray and Fellows, 2022). Some of these BLA projection targets also provide reciprocal inputs. This implies that while the BLA exerts unidirectional control on specific targets, other targets may exert influence on BLA activity as well, but the degree to which the BLA is capable of regulating aspects of its activity through these feedback loops has remained elusive thus far (Figure 1). Future research is needed to understand more about how these feedback circuits are wired through specific cell types and how various feedback mechanisms orchestrate amygdala activity to control behavioral responses.

Recent studies have suggested that the projection targets of the BLA may offer insight into the roles of various projecting populations that originate in the BLA. For example, BLA neurons that project to the NAcc are critical for reward and avoidance learning (Ambroggi et al., 2008; Jones et al., 2010; Pascoli et al., 2011; Stuber et al., 2011; Britt et al., 2012; Namburi et al., 2015; Ramirez F. et al., 2015; Ramirez S. et al., 2015; Zhang X. et al., 2021), while those that project to the CeA are critical for fear (Jimenez and Maren, 2009; Namburi et al., 2015; Kim et al., 2016). However, differential valence processing may exist even within the same pathway as reward and aversion activated neurons differentially engage CeA circuits (Kim et al., 2017) and control different compartments of ventral striatal circuitry (Zhang X. et al., 2021; Figure 1). Further, the encoding of motivationally-relevant behaviors by the BLA may also be influenced by anterior-posterior gradients in reward and aversion neurons in the BLA and their outputs (Kim et al., 2016; Beyeler et al., 2018). Recently, the ITCs of the amygdala have been shown to inhibit BLA output neurons to the prelimbic and infralimbic prefrontal cortex, which are involved in fear acquisition and extinction, respectively (Hagihara et al., 2021). This not only indicates that the BLA encodes valence but also suggests that an understanding of the efferent and topographical organization of the BLA may help to resolve its involvement in various behaviors, offering a greater lens into how the BLA may be engaged in both appetitive and aversive tasks.

### Central amygdala inputs and outputs

Unlike the BLA, the CeA receives few cortical inputs apart from the insular cortex (Kargl et al., 2020), with virtually non-existent outputs of the CeA to cortical areas. The CeA receives afferent inputs from various limbic regions including the BLA, thalamus, BNST, and the ITCs (Royer et al., 1999; Bienkowski and Rinaman, 2013; Kim et al., 2017). Most output pathways of the CeA arise from the CeM and send inhibitory projections to various brainstem regions as well as the hypothalamus, periaqueductal gray (PAG), substantia nigra/ventral tegmental area, BNST, PVT, and parabrachial nucleus (PBN) (Pape and Pare, 2010; Penzo et al., 2014; Gilpin et al., 2015; Douglass et al., 2017; Ahrens et al., 2018; Baumgartner et al., 2021; Borrego et al., 2022). These outputs are capable of rapidly modulating defensive behaviors in response to threats, approach behavior, and reward-related responses. Work has also demonstrated that CeA projection neurons are involved in appetitive behaviors. GABAergic serotonin receptor 2a (Htr2a)-expressing CeA neurons modulate food consumption in mice (Douglass et al., 2017), while CRF-expressing CeA neurons are involved in the motivation to consume rewards and their activation enhances the recruitment of brain areas involved in motivation and reward (Calu et al., 2010; Steinberg et al., 2020; Warlow and Berridge, 2021; Figure 1).

### Subpopulations of basolateral amygdala cell types

The BLA and CeA structures are quite different in terms of their cellular composition. The BLA bears features largely similar to cortical structures (Carlsen and Heimer, 1988). The BLA primarily contains cortical neuron types, with glutamatergic cells comprising approximately 80% of the total neurons in the BLA and GABAergic interneurons making up the residual 20% (Pape and Pare, 2010). Genetic profiling techniques have revealed that multiple subpopulations of BLA principal neurons differentially contribute to valence encoding. For example, subpopulations of principal neurons expressing Rspo2 and Fezf2 have been shown to contribute to aversive behaviors, while neurons expressing Ppp1r1b promote appetitive behaviors (Kim et al., 2016; Rovira-Esteban et al., 2019; Zhang X. et al., 2021). Anatomical arrangement from dorsoventral/anteroposterior in BLA may in part contribute to the encoding of positive or negative valence (Kim et al., 2016; Beyeler et al., 2018; Figure 1).

The GABAergic population of BLA neurons consists of many of the same interneuron subtypes found in cortical regions, with distinct subpopulations of interneurons positive for the calcium-binding proteins parvalbumin, calbindin, and calretinin, along with neuropeptides including vasoactive intestinal peptide (VIP), somatostatin (SOM), cholecystokinin (CCK), among others. These various interneuron subtypes each play a distinct role in the modulation of BLA excitability and synaptic integration. As peptides diffuse over larger areas than amino acid neurotransmitters (Nassel, 2009), the peptidergic interneurons of the BLA are thought to provide modulatory inputs to local interneurons and principal neurons alike. Many studies have examined the involvement of BLA peptidergic neurons in behavior, although very few of these have probed the roles of the various peptides themselves in these functions (Mascagni and McDonald, 2003; Krabbe et al., 2019).

**FIGURE 2:**
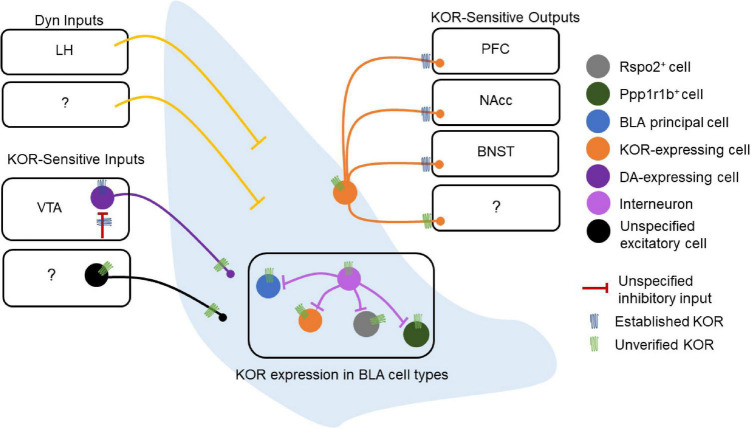
Dynorphin/kappa opioid receptor expression in basolateral amygdala (BLA) circuitry. Schematic of the experimentally-established components of the BLA Dyn/KOR system [e.g., verified kappa opioid receptors (KORs)] as well as some outstanding questions (e.g., unverified KORs). The lateral hypothalamus (LH) is the only structure currently reported to send Dyn inputs to the BLA, while other potential sources of Dyn to the BLA remain to be discovered. Other inputs to the BLA are regulated by the Dyn/KOR system as well. The VTA expresses KORs at DAergic cell bodies, and these same cell bodies are regulated by an inhibitory input that expresses KOR presynaptically, suggesting that the Dyn/KOR system may modulate the release of other neuromodulators such as DA into the BLA. These VTA inputs, as well as other potential inputs to the BLA, may also express KORs presynaptically. The BLA does not contain Dyn neurons, and the cell type-specific expression of KOR in BLA neurons is also poorly understood. Similarly, whether local BLA interneurons express KOR, and, if so, whether KOR^+^ and KOR^–^ interneurons differentially regulate different populations of BLA neurons is unknown. KOR^+^ BLA principal neurons target the PFC, NAcc, and BNST, other projection targets of KOR-expressing neurons remain to be discovered. Likewise, whether KOR is present at somatodendritic compartments or regulates local collaterals within the BLA is not known.

Kappa opioid receptors (KORs) are expressed in BLA neurons, providing a means for Dyn inputs to the BLA to regulate BLA microcircuit function (Figure 2). However, the role of the Dyn/KOR system within BLA microcircuits that control input-output transformations remains largely unresolved. As such, the precise BLA circuits and cell types through which this system regulates behavior must be clarified in greater detail.

### Central amygdala cell types

The cell types and circuits of the CeA bear more resemblance to the inhibitory neurons of striatal circuits, in contrast to primarily excitatory cell types seen in the BLA. Most CeA neurons express GAD65 or GAD67, with very few expressing vGlut1 or vGlut2 (Poulin et al., 2008). Like the striatum, the CeA is embryonically derived from the lateral ganglionic eminence (Swanson and Petrovich, 1998). The CeA and striatum are also characterized by similar expression patterns of cell fate markers (Medina et al., 2011) [see Aerts and Seuntjens (2021) for a recent review on amygdala development].

Neurons in the lateral region of the CeA (CeL) are often categorized by expression, or lack thereof, of the delta isoform of protein kinase C (PKCδ) (Haubensak et al., 2010). PKCδ^+^ neurons in the CeL also tend to express oxytocin receptors and are inhibited during fear states. Furthermore, this population inhibits PKCδ^–^ neurons in the CeL, although PKCδ^+^ and PKCδ^–^ neurons alike project to the CeM. The peptide SOM is also expressed in the CeA, primarily in the CeL (Li et al., 2013; Kim et al., 2017). Here it is important to note that the properties and functions of these CeA SOM cells differ from the SOM cells found in the BLA. Furthermore, the CeL SOM and PKCδ^+^ populations are distinct, with very little overlap (Li et al., 2013; Kim et al., 2017; Wilson et al., 2019). Owing to the differential functions of these populations, CeA SOM and PKCδ^+^ neurons have a role in the consolidation of differential threat memories in CeL, inhibition of SOM or PKCδ^+^ interneurons impaired the time the animals freeze to a threat and safety responses (Wilson et al., 2019; Shrestha et al., 2020; Figure 1).

Central amygdala (CeA) GABAergic neurons co-express mRNA of several peptides including SOM, enkephalin, CRF, neurotensin, and tachykinin (Day et al., 1999). Dyn-expressing neurons are located primarily in the CeL and CeM subregions of the CeA (Kim et al., 2017). Populations of CeA neurons impact the function of the HPA axis (Buller et al., 2001). In mice, the BLA-CeA circuit regulates anxiety-like behavior (Tye et al., 2011), and the knockdown of CRF in CeA neurons reduces anxiety-like behavior (Pomrenze et al., 2019; Ventura-Silva et al., 2020). As such, it is thought that chronic stress produces changes that remodel amygdala circuitry, which can negatively impact performance on cognitive tasks (Roozendaal et al., 2009; Cacciaglia et al., 2017; de Quervain et al., 2017). Conditioned fear responses driven by CeM neurons, presumably PKCδ^+^ neurons, disinhibit CeL output neurons for fear acquisition (Ciocchi et al., 2010; Figure 1).

## Dynorphin/kappa opioid receptor in amygdala circuits

### Basolateral amygdala

Dynorphin/kappa opioid receptor signaling may shape synaptic transmission in BLA circuits. KOR mRNA expression and protein immunoreactivity in the BLA has been described in several studies since the 1990s (DePaoli et al., 1994; Knoll et al., 2011; Van’t Veer et al., 2013; Tejeda et al., 2017; Maiya et al., 2021), while Dyn-expressing neurons are largely absent in the BLA. In humans, prodynorphin mRNA expression is observed in the amygdalohippocampal and accessory basal nuclei, and this expression is reduced in patients with major depressive disorder or bipolar disorder (Hurd, 2002). Pdyn mRNA in the BLA is generally not observed in mice but has been reported in lateral ITCs (Gomes et al., 2020). The KOR agonist U50 reduces excitatory synaptic transmission (as assessed by field EPSPs) in the BLA and blocks high frequency stimulation-induced long-term potentiation of excitatory synapses (Huge et al., 2009). However, in another study examining glutamatergic transmission onto BLA pyramidal neurons with spontaneous excitatory postsynaptic currents using whole-cell slice electrophysiology, the KOR agonist U69,593 was without effect (Przybysz et al., 2017). These inconsistent findings may result from differential sampling of excitatory synapses as the former study examined fEPSPs evoked by LA stimulation, while the latter study was agnostic to the source of excitatory synapses. In contrast, GABAergic sIPSC frequency, but not amplitude, was increased by KOR agonists (U69 and Dyn) in the BLA of adolescent, but not adult, rat brain slices (Przybysz et al., 2017). The U69 effect on sIPSC frequency was blocked by the KOR antagonist nor-BNI and TTX bath application, which indicates that this effect was KOR-dependent and is consistent with a mechanism that acts on action potential-dependent inhibitory transmission and/or indirect circuit-level mechanisms. These results suggest that KORs may act to limit synaptic transmission within BLA circuits. However, from these studies, it is unclear whether KORs act on BLA principal neurons, interneurons, or KOR-expressing afferent inputs to the BLA. KOR in BLA neurons also regulates their outputs to downstream targets (Tejeda et al., 2013, 2015; Crowley et al., 2016). KORs are expressed in BLA terminals innervating the NAcc where they preferentially inhibit glutamate release onto D1 vs. D2 MSNs (Tejeda et al., 2017). KORs also inhibit the release of glutamate from BLA terminals in the mPFC and the BNST (Tejeda et al., 2015; Crowley et al., 2016; Figure 2). These studies suggest that Dyn released within BLA target regions, such as the NAcc, mPFC, and BNST, may modulate glutamatergic inputs from BLA neurons, decoupling BLA terminal control of target cells without inhibiting the BLA projection neuron at the soma. KORs preferentially inhibit inputs to NAcc D1 versus D2 MSNs (Tejeda et al., 2017), raising the possibility that either KORs are trafficked to specific BLA terminals based on their postsynaptic targets and/or that KOR-containing and KOR-lacking BLA projecting neurons differentially innervate D1 and D2 MSNs. It is unclear whether KOR regulation of BLA outputs onto molecularly-or projection-defined targets in other BLA terminal brain regions differs, such as the BNST and mPFC (Figure 2). Together, these studies demonstrate that the Dyn/KOR system can regulate inhibition and excitation onto BLA neurons and their outputs to target structures.

### Central amygdala

**FIGURE 3:**
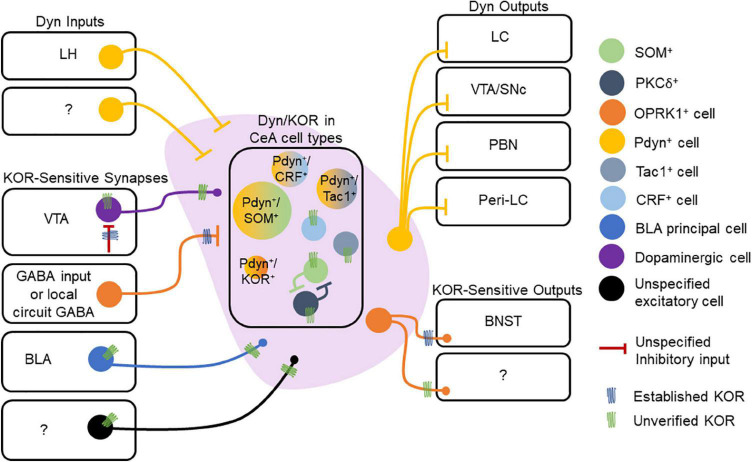
Dynorphin/kappa opioid receptor expression in central amygdala (CeA) circuitry. Dyn is expressed in multiple cell types within the CeA. Extrinsic Dyn inputs to the CeA arise from lateral hypothalamus (LH), but other sources remain unknown. Presynaptic KOR regulation of inhibitory synapses onto CeA neurons has documented, but the source of KOR + GABA neurons is not known. Other inputs to the CeA may also express KOR, including those arising from the VTA, basolateral amygdala (BLA), and other uncharacterized regions. Within the CeA, KOR expression within specific cell types is not understood. KORs may directly hyperpolarize or inhibit collaterals established by CeA neurons. CeA projections to the BNST are inhibited by presynaptic KORs. KOR expression at other CeA neuron outputs has not been demonstrated. CeA Dyn neurons are known to project to multiple regions including the LC, VTA/SNc, PBN, and Peri-LC. The molecular profiles of Dyn neurons may also vary across the CeA’s medial and lateral subdivisions, which we are omitted in the figure.

In contrast to the BLA, neurons of the CeA express Dyn. *Pdyn* and *Oprk1* mRNA expressing cells are primarily non-overlapping populations in the CeA, with a smaller subset of cells expressing both *Pdyn* and *Oprk1* mRNA (Bloodgood et al., 2021). Dyn is also co-expressed with other neuropeptides within the CeA. Nearly all Dyn-expressing neurons in the CeL co-express proenkephalin (Penk), SOM, and Tac2 mRNA (Kim et al., 2017), and approximately 80% of Dyn-expressing CeL neurons co-express SOM peptide (Jungling et al., 2015). About 35% of Penk-expressing neurons in CeL co-express dynorphin (Kim et al., 2017). In the CeM, nearly all Dyn-expressing neurons co-express the dopamine receptor Drd1a, and nearly all Tac1 neurons co-express Dyn (Kim et al., 2017), consistent with Dyn expression patterns observed in striatal cells expressing Tac1 and Drd1a. Furthermore, a subset of GABAergic CeL neurons co-expresses CRF and prodynorphin (Marchant et al., 2007; Kim et al., 2017; Sanford et al., 2017; Figure 3). Collectively, these results suggest that Dyn-expression is embedded in CeA circuits in a sub-region and cell-specific manner. Given that different molecularly defined CeA cells and sub-regions play fundamentally different roles in motivationally-charged behaviors then Dyn in distinct cell types and sub-regions is hypothesized to differentially control behavior and circuit function. Like we mentioned in the prior section, it will be important for human postmortem studies to investigate the distribution of the Dyn/KOR system among populations of CeA neurons and contrast these with the established circuitry of mice.

Dynorphin acting within the CeA regulates inhibitory synaptic transmission. KOR activation inhibits GABA release onto CeA cells *via* a presynaptic site of action (Kang-Park et al., 2013, 2015; Gilpin et al., 2014; Hein et al., 2021). These results suggest that Dyn may decrease inhibition of CeA cells and contribute to the disinhibition of CeA circuits. Approximately half of CeA neurons characterized by strong spike accommodation and lack of an after-depolarization potential (ADP) are hyperpolarized by the KOR agonist U69,593, while cells lacking spike accommodation and with ADPs were insensitive to U69,593 but directly hyperpolarized by met-enkephalin (Zhu and Pan, 2004). These results demonstrate that Dyn/KOR signaling may also directly hyperpolarize subsets of cells in the CeA. Given that intrinsic firing properties differ between CeA cell types (Zhu and Pan, 2004; Haubensak et al., 2010; Wilson et al., 2019; Adke et al., 2021), these results suggest that Dyn may decrease the excitability of subsets of CeA cells *via* actions at somatodendritic KORs. Further, CeA cells form local collaterals within CeA circuits (Cassell et al., 1999), and as such, it is possible that pools of KOR-sensitive inhibitory synapses may arise from within the CeA. KORs inhibit glutamatergic transmission of electrically evoked glutamatergic transmission in the BLA to subsets of CeL neurons (Kissiwaa et al., 2020), but fails to modify PBN to CeL synapses (Kissiwaa et al., 2020; Hein et al., 2021; Figure 3). These results suggest that Dyn released from CeA neurons may regulate local circuit inhibition and incoming afferent inputs in a pathway-specific manner.

Central amygdala Dyn neurons target several regions involved in reward-, fear-, and stress-related behaviors. The CeA sends a Dynergic projection to the locus coeruleus (LC), and about 42% of those projection neurons co-express CRF and Dyn (Reyes et al., 2011). Dyn-expressing CeA neurons are modulated by LC neurons, specifically those expressing NE (Kravets et al., 2015). Furthermore, approximately 30% of these NE-expressing terminals from LC target CeA neurons that co-express CRF and Dyn. Dynergic CeL neurons project to the LC, and approximately 50% of the CeL inputs to the LC express both Dyn and SOM (Jungling et al., 2015). Additionally, less than 3% of the PKCδ^–^ expressing CeL neurons that project to peri-LC are positive for Dyn, suggesting that PKCδ and Dyn populations are largely non-overlapping (Jungling et al., 2015). In addition to the LC, Dyn CeA neurons also project to the PBN. Approximately 20% of retrograde fluorogold-labeled neurons in CeA that project to the PBN are Dyn^+^, and another 15% co-express Dyn and SOM (Raver et al., 2020), suggesting that co-expression of Dyn and SOM may differ between CeA outputs (Figure 3). The CeA pathway is inhibited in mouse models of chronic pain, and stimulation of this pathway blocks pain-related behavioral phenotypes, providing a potential circuit-based mechanism by which CeA Dyn neurons may influence nociceptive and affective behaviors driven by pain states. PDyn-and SOM-expressing neurons in the CeA also project to the substantia nigra (Steinberg et al., 2020), thus targeting a key brain region involved in appetitive behaviors and motivational drive.

External inputs of Dyn to the CeA were documented by Zardetto-Smith et al. (1988) who reported the presence of Dyn-expressing neurons in the LH and perifornical nucleus that project to the CeA of rats. Orexinergic neurons from the LH also innervate the CeA, and about 95% of orexinergic neurons express dynorphin (Peyron et al., 1998; Chou et al., 2001). KORs also regulate CeA projections *via* inhibition of presynaptic GABA release in downstream targets. For example, application of KOR agonists reduced GABAergic transmission onto BNST neurons from CeA afferents (Li et al., 2012). Dyn released from BNST neurons activates presynaptic KORs located on CeA afferents to inhibit GABAergic transmission to the BNST and opposes the facilitatory effect of endogenously released neurotensin from BNST neurons on CeA GABA inputs (Normandeau et al., 2018). These results suggest that Dyn released from BNST neurons may act as a retrograde signal to limit KOR-sensitive inputs. Dynergic tone on inhibitory synapses in the BNST, which may potentially arise from CeA SOM-expressing CeA neurons, is enhanced in stressed mice and mice lacking ErbB4 in SOM-positive neurons (Ahrens et al., 2018; Figure 3). Collectively, these results suggest that Dyn/KOR is integrated within CeA circuits and regulates CeA local circuits and outputs. Taken together, robust Dyn/KOR expression and regulation of amygdala circuit function position this system to regulate amygdala-dependent control of motivationally charged behaviors and experience-dependent appetitive and aversive learning.

## Stress and amygdala dynorphin/kappa opioid receptor signaling

The stress response plays a key role in the ability of organisms to adapt following exposure to threatening stimuli or experiences (LeDoux, 2000). CRF plays a significant role in the integration of endocrine and behavioral responses to stress (Vale et al., 1981). CRF is released as a neurotransmitter from neurons in the CeA and BNST and is secreted as a neurohormone from PVT neurons to induce the secretion of adrenocorticotropic hormone (ACTH) from the anterior pituitary gland. ACTH enters the bloodstream to access the adrenal gland cortex, where it stimulates the secretion of glucocorticoids, initiating the systemic stress response (Dedic et al., 2018). Importantly, glucocorticoids can regulate gene expression by binding glucocorticoid receptors (GR) (Meijsing, 2015). Dyn cells in the central amygdaloid nucleus contain GR immunoreactivity (Cintra et al., 1991), suggesting that glucocorticoids may regulate Dyn expression in CeA as they do in other brain regions like the hippocampus (Thai et al., 1992). Furthermore, early life stress may increase GR binding to the second intron of OPRK1, the gene that encodes KOR expression, to influence gene expression (Lutz et al., 2018). These studies suggest that stressor-driven fluctuations in CRF and glucocorticoids may modulate the Dyn/KOR system in the amygdala to regulate behavior. Since stress modalities and durations are diverse, different stress-related signaling molecules and defensive behaviors may be mobilized to promote resilience or susceptibility. Therefore, future work further dissecting how the Dyn/KOR system is engaged or regulates different forms of stress is imperative for furthering our understanding.

### Basolateral amygdala

Psychological stress increases Dyn/KOR activity in the BLA. Dyn/KOR activation is necessary for a variety of stress-related and aversive behaviors. Interestingly, acute forced swim stress and CRF both increase phosphorylation of KOR (a putative index of KOR activity) in the BLA, but not in the CeA (Land et al., 2008). Dyn expression is also increased in the BLA of male mice following 14 days of social defeat stress (Zan et al., 2022). Moreover, systemic KOR antagonists abolish the increase of phosphorylation of ERK after social defeat stress in the BLA, suggesting that KOR signaling is potentially upstream of ERK induction. KOR agonist decreases GABAergic transmission in most BLA neurons from stressed adolescent male mice relative to unstressed controls (Varlinskaya et al., 2020), suggesting that stress may impact KOR regulation of inhibitory transmission. Increased BLA KOR phosphorylation induced by CRF injection is similarly blocked by KOR antagonism (Bruchas et al., 2009; Knoll et al., 2011). BLA KORs also interact with stress-related signaling pathways to drive the expression of stress-induced nicotine drug reinstatement (Nygard et al., 2016). Together, these findings highlight that activation of the BLA Dyn/KOR system during stress may impact the function of the BLA in regulating stress-associated behavioral responses. Given the central role of stress in fear and anxiety, it is also important to cover the role of the BLA Dyn/KOR system in these states.

Fear-related behaviors in response to threats are, by definition, driven by aversion, and many signaling pathways associated with stress are often similarly engaged during fear states. As such, the Dyn/KOR system in the BLA is well-suited to contribute to the formation and/or maintenance of fear memory. In rats, KOR mRNA in the BLA increases with conditioning with fear-potentiated startle (FPS) and decreases with extinction of FPS (Knoll et al., 2011). Microinjection of the KOR antagonist JDTic into the BLA of rats reduces fear expression. In Dyn KO mice, levels of c-fos protein in the BLA are decreased relative to WT controls in response to fear extinction but not during fear conditioning to auditory cue and footshock pairings (Bilkei-Gorzo et al., 2012). These studies imply not only that fear expression is associated with changes in Dyn/KOR signaling in the BLA, but also that the Dyn/KOR system mediates fear behavior by acting within the BLA.

Similarly, activation of the BLA Dyn/KOR system during stress may serve to regulate depressive-or anhedonia-like states that persist after exposure to a stressor. BLA activity, as indexed by c-fos, is reduced in Dyn KO mice relative to controls following exposure to the anxiogenic zero maze (Bilkei-Gorzo et al., 2008), suggesting that loss of Dyn/KOR signaling reduces BLA neuronal activation. However, it is important to note that this study did not provide measurements of BLA c-fos from naive animals not exposed to the zero maze. Microinjection of KOR antagonists in the BLA increases the time in the interaction zone after social defeat stress and prevents the development of depressive-like behaviors induced by chronic social defeat stress (Zan et al., 2022). Intra-BLA administration of a KOR antagonist produces anxiolytic-like effects in the elevated plus maze in rats (Knoll et al., 2011). Consistent with the hypothesis that BLA KOR is involved in anxiety-related behavior, deletion of KOR from the BLA of adult male mice results in more time in the open arms of the elevated plus maze, suggesting that loss of BLA KOR function may confer an anxiolytic effect (Crowley et al., 2016). Microinjections of the KOR antagonist nor-BNI into the BLA reduce anxiety-like behavior following acute stress exposure or CRF administration in mice (Bruchas et al., 2009). Together, these results demonstrate that Dyn/KOR signaling within the BLA may be engaged to promote innate anxiety-like behavior and learned fear.

### Central amygdala

The CeA Dyn/KOR system has also been studied in the context of stress responsivity. Forced swim stress increases dynorphin expression in the CeA of mice following stress exposure (Chung et al., 2014). Another study showed that unescapable tail shock increased DynA (1–8) immunoreactivity in the anterior portion of the lateral CeA (Gouty et al., 2021). In CeA, CRF, a neuropeptide that is a critical mediator of the stress response, is co-expressed with Dyn (Reyes et al., 2008; Kravets et al., 2015) and Dyn KO mice have been shown to have reduced expression of CRF in CeA (Wittmann et al., 2009). Activation of KORs in the CeA with U69,593 drives aversion and anxiety-like behavior, and this effect can be blocked by optogenetic inhibition of CeA CRF neurons. KORs disinhibit CeA CRF neurons *via* inhibition of presynaptic GABA release and feed forward inhibition driven by the PBN onto CeA CRF neurons (Hein et al., 2021). These results suggest that CeA Dyn/KOR signaling may modulate CRF neuron activity and CRF peptidergic transmission, which would influence defensive responses to stressors and anxiety-related behavior under the control of CeA CRF neurons (Fadok et al., 2017; Sanford et al., 2017).

Loss of KORs in the CeA, as well as loss of Dyn inputs to the CeA, increases anxiety-like behavior and promotes fear generalization wherein defensive freezing is observed in response to both threat-predictive cues and neutral cues alike (Baird et al., 2021), suggesting that Dyn released from CeA neurons may limit excessive passive defensive behaviors to non-threatening experiences and environmental cues. In ErbB4 gene deficiency mice, the Dynergic activity of SOM-expressing CeA inputs to BNST also promotes anxiety-like behavior *via* a disinhibitory effect (Ahrens et al., 2018). Likewise, Dyn knockdown in CRF-expressing CeA neurons reduces anxiety-like behavior as evidenced by increased exploration of open arms in the EPM as well as increased time in the center of the OFT (Pomrenze et al., 2019). Fear conditioning and fear retrieval increase pCREB in Dyn-expressing CeL neurons, relative to both naive home cage mice as well as mice that received unpaired CS/US delivery (Jungling et al., 2015), suggesting that CeA Dyn neurons may not be solely engaged by threats, but rather cues that predict threats.

## Regulation of drug and alcohol seeking behavior by amygdala dynorphin/kappa opioid receptor neurons

### The basolateral amygdala dynorphin/kappa opioid receptor system modulates drug-seeking behavior

The BLA Dyn/KOR system has also been studied in the context of alcohol and nicotine seeking behavior, where it has been implicated in playing a role as a negative reinforcer that promotes drug-seeking behavior aimed at curbing drug withdrawal-induced and stress-induced negative affect and/or anhedonia which maintain drug-seeking behavior (Koob et al., 2014). Male constitutive Dyn-KO mice have a higher preference for alcohol consumption relative to WT controls, and footshock increases alcohol consumption in WT mice but not Dyn-KO mice (Racz et al., 2013). Furthermore, mild footshock following chronic alcohol exposure increases BLA c-fos levels in WT mice but reduces BLA c-fos in Dyn-KO mice (Racz et al., 2013). A more recent study identified the KOR-encoding gene OPRK1 as a target of the transcriptional regulator LMO4 (Maiya et al., 2021). The authors also found that U50,488-mediated increases in alcohol consumption are attenuated in LMO4-shRNA*^BLA^* mice and that local infusion of the KOR antagonist nor-BNI in the BLA reduces alcohol consumption in mice. The role of Dyn/KOR signaling in mediating the reinforcing properties of other drugs has not been thoroughly investigated. KOR antagonism during reinstatement of stress-induced nicotine conditioned place preference (CPP) reduces c-fos expression in the BLA and deletion of KOR from BLA neurons blocks reinstatement of stress-induced nicotine CPP (Nygard et al., 2016). The finding that nor-BNI blocks stress-induced reinstatement effects on BLA c-fos immunoreactivity suggests that the function of KORs in the BLA is more complex than simply blocking the inhibitory actions of KOR signaling. Similar effects of KOR antagonism on stress-induced nicotine-seeking behavior were observed in a separate study, although the specific amygdala subregion studied was not delineated (Smith et al., 2012). DREADD-mediated activation of G_*ai*_ signaling, an inhibitory signaling pathway that is known to be activated by KORs, in BLA PNs is also sufficient to drive this reinstatement effect (Nygard et al., 2016). These studies demonstrate that Dyn/KOR signaling specifically in the BLA regulates negative reinforcement processes that drive drug- and alcohol-seeking behaviors.

### The central amygdala dynorphin/kappa opioid receptor system modulates the consumption of alcohol, psychostimulants, and opioids

Repeated alcohol exposure produces maladaptive behavioral effects which in part are hypothesized to be mediated by Dyn/KOR signaling in the CeA (Pohorecky et al., 1989; Dar, 1998; Matsuzawa et al., 1999; Walker and Kissler, 2013; Kissler et al., 2014; Anderson et al., 2018). Alcohol consumption increases the expression of both Dyn and KOR in the amygdala, including the CeA and BLA (D’Addario et al., 2013). In a rat model of alcohol dependency induced by ethanol vapor exposure, dependent rats displayed increased Dyn immunoreactivity and functional KOR coupling to G-proteins in the amygdala (Kissler et al., 2014). However, binge alcohol drinking in the drinking in the dark paradigm did not result in changes in the expression of Pdyn and Oprk1 mRNA in the CeA of mice (Bloodgood et al., 2021). An *in vivo* microdialysis study also reported that extracellular Dyn peptide levels in the CeA are increased following high doses of ethanol associated with intoxication (Lam et al., 2008). These studies highlight that Dyn expression and release may be recruited by intoxicating or dependence-producing ethanol exposure. In slices, KOR activation by dynorphin impairs ethanol-induced increases in IPSP amplitude in the CeA, while KOR antagonism with nor-BNI increases CeA IPSPs (Gilpin et al., 2014), suggesting that alcohol increases the activity of KOR-mediated inhibitory synapses. Alcohol drinking drives sex-specific effects on the excitability of Pdyn-expressing neurons in the CeA without impacting excitatory synaptic drive onto these neurons (Bloodgood et al., 2021). Together, these studies indicate that ethanol consumption promotes Dyn/KOR signaling in the CeA. Consistent with the hypothesis that enhanced CeA Dyn promotes negative reinforcement of alcohol seeking behavior, inhibition of the Dyn/KOR signaling pathway in the CeA reduces alcohol consumption. Deletion of *Pdyn* decreases ethanol drinking in both male and female mice, while CeA *Oprk1* ablation in reduces alcohol-seeking in males but not females (Bloodgood et al., 2021). Administration of the KOR antagonist nor-BNI in the CeA reduces ethanol self-administration across multiple models of alcohol consumption, including a drinking in the dark model and in alcohol-dependent rats (Kissler et al., 2014; Kissler and Walker, 2016; Anderson et al., 2019). Intra-CeA nor-BNI also reduces ethanol self-administration during acute withdrawal and protracted abstinence, suggesting mitigated psychological sensitivity to withdrawal symptoms, although this surprisingly did not affect physiological measures of alcohol withdrawal (Kissler and Walker, 2016). Finally, alcohol consumption increases Pdyn-mRNA expression in CeA, and inhibition of PDyn-expressing neurons in the CeA or KOR antagonism in the CeA or bed nucleus of the stria terminalis, also reduces alcohol consumption (Anderson et al., 2019; Haun et al., 2022). These behavioral studies underscore a causal role for Dyn/KOR signaling in mediating negative reinforcement underlying compulsive alcohol seeking behavior.

Dynorphin/kappa opioid receptor activity in the CeA is also associated with psychostimulant and opioid seeking behavior. One study found that the KOR antagonist nor-BNI and KOR agonist U50,488 reduced and increased, respectively, GABAergic neurotransmission in the CeA of rats with long access (6 h) to cocaine (Kallupi et al., 2013). Interestingly, in controls, CeA GABAergic transmission was inhibited by KOR activation, suggesting that long access to cocaine inverts KOR regulation of CeA inhibitory synaptic transmission. In this study, CeA KOR antagonism blocked cocaine sensitization, indicating that Dyn/KOR signaling in the CeA may promote incentive salience with repeat cocaine exposure, as well as decreasing anxiety-like behavior during cocaine withdrawal. One study used the chemical stressor yohimbine to drive stress-induced reinstatement of heroin seeking in rats. Here, dynorphin precursor mRNA levels were enhanced in the CeA, but not BLA or medial amygdala, of yohimbine-treated mice, suggesting that CeA Dyn may promote stress-induced heroin-seeking behavior (Zhou et al., 2013). Collectively, these studies demonstrate that Dyn/KOR signaling within the CeA is engaged by various misused substances and this subsequently regulates drug-seeking behavior and ensuing maladaptive behaviors.

## Regulation of pain by amygdala dynorphin/kappa opioid receptor neurons

It is hypothesized that Dyn/KOR signaling in the CeA may mediate aspects of pain processing. In a spinal nerve ligation (SNL) model of pain, nor-BNI in the right CeA blocked conditioned place preference (CPP) driven by gabapentin (an FDA approved treatment for neuropathic pain), suggesting that CeA KOR signaling is necessary for pain-induced negative affect (Navratilova et al., 2019). Further, intra-CeA KOR antagonism blocks anxiety-like behavior and ultasonic vocalizations in a rat functional pain model wherein morphine priming sensitizes stress-induced pain-like and affective behavior (Yakhnitsa et al., 2022). Moreover, increased CeA Dyn signaling may shape pain-related behavior as intra-CeA KOR antagonism blocks defensive behaviors in response to noxious stimuli using Randall Selitto to measure the paw withdrawal threshold (Phelps et al., 2019) or sensitivity to capsaicin left forepaw injection in a functional pain model involving morphine priming in rats exposed to a bright light stimulus (Nation et al., 2018). Furthermore, electrically evoked IPSCs onto CeA neurons are only potentiated by nor-BNI in SNL rats but not sham controls (Navratilova et al., 2019). Moreover, PBN-evoked polysynaptic inhibition or electrically-evoked IPSCs are potentiated by nor-BNI in the functional pain model described above (Yakhnitsa et al., 2022). These studies suggest that heightened Dyn signaling may be contributing to CeA neuron disinhibition in pain states. Disinhibition of CeA neurons by increased Dyn tone facilitates synaptically-evoked spiking in SNL rats, suggesting that Dyn may influence input-output transformations within CeA circuits (Navratilova et al., 2019). Administration of complete Freund’s adjuvant (CFA) in mice increases G-protein stimulation in the CeA with the KOR agonist ICI 199,441 relative to saline-injected control mice (Narita et al., 2006), and Dyn content in the CeA of a functional pain model (Nation et al., 2018). Furthermore, intra-CeA KOR activation with U69,593 potentiates responsivity of amygdala and spinal cord neurons in response to noxious stimuli, an effect that is reversed by optogenetic silencing of CeA CRF neurons (Ji and Neugebauer, 2020). These findings suggest that Dyn disinhibition of CeA CRF neurons is a critical component underlying the effects of Dyn on CeA circuits and control of behavior. Recently, CeA SOM and PKCδ neurons have been shown to be differentially involved in pain regulation, with PKCδ and SOM cells promoting and inhibiting nociceptive responses, respectively (Wilson et al., 2019). Given that CeA SOM neurons robustly respond to threats [reviewed above and in Yu et al. (2016)], it is possible that pain, a strong threat that promotes maladaptive passive defensive behaviors, robustly recruits SOM neurons co-expressing Dyn. Pain-induced recruitment of CeA Dyn neurons would disinhibit incoming afferent inputs to the CeA that process noxious stimuli, including the PBN. The aforementioned hypothesis would address the discrepancy in the field wherein CeA SOM neurons have been widely implicated in mediating fear-related freezing (fear ON cells) and paradoxically inhibit nociceptive responses in animal models of chronic pain (pain OFF cells). However, further work is needed to understand how Dyn may be interacting with distinct cell populations, such as PKCδ, SOM, and CRF neurons, to orchestrate maladaptive behavior induced by CeA dysfunction driven by pain, stress, and/or misused substances.

## Cracking the shell on the almond: A circuit-based framework for amygdala dynorphin/kappa opioid receptor control of behavior

Here we posit that the Dyn/KOR system is poised to orchestrate coordinated waves of inhibition or disinhibition by targeting specific cell types and afferent inputs to amygdala circuits *via* the circuit-specific actions of amygdala Dyn on KOR-expressing neurons. As KOR is a G_i_-coupled receptor, which regulates presynaptic neurotransmitter release or intrinsic excitability, activation of KOR by Dyn will produce fundamentally different outcomes depending on which amygdala cell type and/or afferent input expresses KORs. An obvious major distinction is that the BLA and CeA consists primarily of glutamatergic and GABAergic projection neurons that also collateralize within local circuits. Thus, KOR signaling in excitatory BLA neurons vs. CeA neurons would produce distinct outcomes since divergent circuit motifs would be engaged by the Dyn/KOR system, including decreased excitatory drive or disinhibition, respectively. Within the BLA, for example, activation of KORs on principal neurons would suppress transmission of those neurons to downstream targets such as the CeA. Activation of KORs on GABAergic CeA neurons, meanwhile, would serve to disinhibit downstream targets of those neurons and in efferent regions (Figure 2). Since molecularly- and projection-defined cells in the BLA and CeA in large part account for various aspects of threat and reward processing, future work is needed to further resolve the specificity of KOR expression within amygdala cell types. It is currently unclear whether KOR is widely expressed in different sub-classes of molecularly-defined neurons such as RSPO2, PPP1R1B, and Fezf2 (Kim et al., 2016; Zhang X. et al., 2021; Figure 2). KORs are on various presynaptic terminals of BLA efferents where they inhibit glutamate release in areas including the NAcc, PFC, and BNST, but whether KORs are ubiquitously expressed across all BLA outputs remains to be resolved. Further, it remains unclear whether KORs in the BLA are poised to control microcircuitry within BLA circuits or solely BLA outputs to downstream targets *via* presynaptic inhibition (Figure 2). KORs in the BLA may be expressed solely on excitatory neurons, or potentially in any of the plethora of interneuron populations within the BLA (Figure 2). Further, KORs may regulate excitatory synapses from KOR-expressing BLA principal neurons to other BLA cell types and interneuron populations. Dyn release and subsequent KOR signaling within BLA circuits may depress recurrent excitatory connections between BLA principal neurons or engaged inhibitory circuit motifs by limiting interneuron recruitment or outputs, depending on how KORs are embedded. This information will be critical for understanding how the Dyn/KOR system shapes activity dynamics of BLA projection neurons. Lastly, Dyn expression is spare or absent in the BLA, which raises the question of what specific sources of Dyn for the BLA may be or whether all sources of BLA Dyn confer the same effects on BLA circuit physiology and behavior. A recent study reported expression of Pdyn mRNA in the lateral ITCs raising the interesting possibility that this may be a source of Dyn to BLA circuits (Gomes et al., 2020).

Within the CeA, Dyn expression is primarily concentrated in SOM neurons, but is also expressed in other SOM-negative cell types (Figure 3). Whether these subpopulations of Dyn-expressing neurons are differentially integrated within CeA local circuits and innervate downstream brain regions is not known. Moreover, CeA Dyn neurons may release Dyn locally to regulate local CeA microcircuits that directly or indirectly impacted by KOR signaling. Determining the specific expression profile of KORs in molecularly-defined cell types and CeA projections would be significantly advance our understanding of how the Dyn/KOR system regulates CeA control of behavior (Figure 3). Dyn release from SOM neurons may regulate GABA release from KOR-expressing terminals from defined cell types within the CeA microcircuit, which has been hypothesized to be critical for regulating different aspects of threat-related behaviors (Moscarello and Penzo, 2022). For example, PKCδ^+^ neurons in the CeA may inhibit SOM^+^ neurons *via* GABA release to diminish freezing behavior in response to a threat, and Dyn release from SOM neurons may limit PKCδ^+^-mediated lateral inhibition if these cells express KORs. Therefore, further research into KOR expression on specific cell types will be critical for our understanding of how Dyn signaling regulates activity among amygdala targets. Through specific functional effects on cellular physiology (e.g., regulation of synaptic transmission and excitability), Dyn signaling in specific cell types expressing KOR may forge inter-cellular communication within amygdala circuitry. Such a mechanism, when considered at the population scale, may help determine the selection and deselection of specific subpopulations to form neuronal ensembles within the amygdala whose signal is able to stand out above the noise of the network (Figure 3). This, of course, remains to be tested with rigorous functional studies that examine the activation patterns of Dyn-or KOR-expressing neurons and how their activity influences inputs and outputs of the amygdala during behavior.

The amygdala Dyn/KOR system may also interact with other neuromodulatory systems that influence amygdala dependent behavior. Dyn neurons in the CeA target other brain regions rich in specific neuromodulators. For example, CeA Dyn neurons project to the VTA and substantia nigra, two known dopaminergic hubs (Fallon et al., 1985; Steinberg et al., 2020; Figure 3). Dyn decreases excitability BLA-projecting VTA DA neurons (Ford et al., 2007; Margolis et al., 2008; Baimel et al., 2017) in addition to inhibiting GABAergic transmission onto these cells (Ford et al., 2007; Figure 2). However, whether the Dyn supplied to the VTA that influence nigro- and meso-amygdaloid neurons arises from the CeA or some other Dynergic region remains to be determined. An alternative, but not mutually exclusive possibility, is that dopaminergic terminals in the amygdala express presynaptic KORs similar to what is observed in mesolimbic and mesocortical dopaminergic pathways (Chefer et al., 2013; Tejeda et al., 2013; Figure 2). If KORs regulate presynaptic DA terminals in amygdala circuitry it would provide a mechanism for Dyn inputs to the BLA or CeA Dyn-expressing neurons to regulate incoming DA inputs which are critical for aversive and reward learning. Amygdala Dyn/KOR signaling may also influence other monoamines. CeA Dyn neurons project to the norepinephrine-rich LC (Kravets et al., 2015), which in turn influences amygdala circuitry and distributed networks (Figure 3). However, how the Dyn/KOR system regulates the norepinephrine system during various behaviors is unknown. In CeA, Dyn expression highly overlaps with SOM, another neuropeptide. Although Dyn and SOM are often expressed in the same CeA neurons (Jungling et al., 2015), much remains unknown about the implications of this co-expression. It is unclear what the behavioral effects of SOM neuropeptide transmission is on affective and motivated behaviors. Further, it is unknown whether Dyn and SOM are differentially released during behavior, and, if so, whether they exert complementary or antagonistic effects on amygdala microcircuitry. It is possible that differences in expression patterns of KOR and the SOM receptor on distinct CeA cell types may confer another layer of complexity by which these two peptides engage or disengage discrete microcircuits. Together, these results highlight the importance of understanding the way the Dyn/KOR system may interact with other neuromodulators in amygdala circuits to influence behavior.

Future efforts should be aimed at investigating how the compartmentalization of the Dyn/KOR system within the amygdala shapes different aspects of amygdala-dependent behavior. Though tremendous progress has been made in understanding the role of amygdala circuitry in controlling associative learning, primarily threat conditioning, over the last couple of decades it has become increasingly clear that the amygdala as a whole regulates many nuanced facets of emotion, goal-directed behavior, and motivation. Through the selection of distinct cell types and circuit motifs, the Dyn/KOR system may aid in mediating specific behavioral outcomes, including associative learning. The Pearce-Hall learning model posits that attention and salience are also critical factors underpinning associative learning, and a growing body of work underscores the role of the amygdala in regulating attentional processing (Roesch et al., 2012). Furthermore, given the restriction of Dyn expression to largely PKCδ^–^ neurons, which are thought to be engaged during fear (Ciocchi et al., 2010; Haubensak et al., 2010), the Dyn/KOR system in the amygdala may regulate valence processing. Therefore, understanding whether the amygdala Dyn/KOR system may be regulating appetitive associative and instrumental behavior is needed to further understand how cells expressing Dyn and/or KOR may be involved in learning processes. Because dopamine in the amygdala is critical for appropriate associative learning (Jo et al., 2018; Lutas et al., 2019). Therefore, in addition to the direct actions of Dyn/KOR signaling on amygdala neurons, the potential for Dyn to inhibit KOR-expressing dopaminergic neurons that project to the amygdala could constitute a mechanism that regulates learning. As models of BLA and CeA circuit function are refined and projection-and molecularly-defined neurons are characterized in terms of how their activity is explained by different learning models, we will be able to place activity of amygdala Dyn neurons or KOR-expressing cells in the context of neural correlates that adhere to different leaning models, such as the Pearce-Hall learning model (Roesch et al., 2012). Moreover, manipulating Dyn/KOR signaling (e.g., pharmacologically, genetically, etc.,) and monitoring the activity of amygdala circuitry broadly in the context of molecular markers and/or connectivity will be essential for determining the role this system plays in learning and cognitive processes by shaping amygdala circuit dynamics. Activation of the Dyn/KOR system by stressors, in conjunction with its ability to itself drive aspects of the stress response, raises the possibility that the Dyn/KOR system may be an important neuromodulator at the interface between an organism’s internal state and external events and stimuli essential for guiding behavior. The amygdala receives significant inputs from structures that incorporate interoception, environmental features, flexible behavior, and action selection, including various prefrontal cortical and neuromodulatory factors such as dopamine to ultimately influence overall behavioral states (Grundemann et al., 2019; Courtin et al., 2022). To summarize, several lines of research are needed in order to piece together how the Dyn/KOR system in the amygdala functions to shape the selection/deselection of distinct cell types, microcircuits, and pathways couple with internal states to regulate complex innate and learned behaviors.

## Conclusion

In conclusion, here we provide an overview of the literature on the amygdala Dyn/KOR system (Figures 2, 3). Despite the considerable evidence that implicates the Dyn/KOR system in the amygdala complex in promoting threat reactivity, chronic pain, and negative reinforcement in models of alcohol and substance use disorder, there is still a major gap in our understanding of the Dyn/KOR system in the amygdaloid nuclei. We identify unknowns and provide a framework that places the function of the Dyn/KOR system in the context of the recent advancements in identifying the role of specific cell types and incoming and outgoing pathways of the amygdaloid complex (Figures 2, 3). This model will also provide general principles that are shared or distinct across neuropeptide signaling in amygdala circuits and the brain. A better understanding of this system will be invaluable in identifying how the Dyn/KOR systems regulate information processing in amygdala circuits and behaviors related to motivation. Additionally, uncovering novel potential targets and translational work will help elucidate new treatments for neuropsychiatric disorders and provide potential mechanisms for targets currently in clinical trials.

## Author contributions

All authors listed have made a substantial, direct, and intellectual contribution to the work, and approved it for publication.
